# Di-μ-benzoato-κ^3^ *O*,*O*′:*O*;κ^3^ *O*:*O*,*O*′-bis­[(benzoato-κ^2^ *O*,*O*′)(1,10-phenanthroline-κ^2^ *N*,*N*′)lead(II)]

**DOI:** 10.1107/S160053681101840X

**Published:** 2011-05-20

**Authors:** Hong-Jin Li, Zhu-Qing Gao, Jin-Zhong Gu

**Affiliations:** a School of Chemistry and Biology Engineering, Taiyuan University of Science and Technology, Taiyuan 030021, People’s Republic of China; b College of Chemistry and Chemical Engineering, Lanzhou University, Lanzhou 730000, People’s Republic of China

## Abstract

In the centrosymmetric dinuclear title compound, [Pb_2_(C_7_H_5_O_2_)_4_(C_12_H_8_N_2_)_2_], two Pb^2+^ ions are connected by two tridentate bridging benzoate anions. The Pb^2+^ ion is seven-coordinated by five O atoms from three benzoate anions and two N atoms from the 1,10-phenanthroline ligands. The benzoate anions adopt two different coordination modes, one bidentate–chelating and one tridentate bridging–chelating. The three-dimensional supra­molecular framework is achieved by inter­molecular π–π stacking inter­actions, with a shortest centroid–centroid distance of 3.617 (4) Å.

## Related literature

For bond lengths and angles in other lead(II) compounds, see: Fan *et al.* (2006 ▶); Hu *et al.* (2011 ▶).
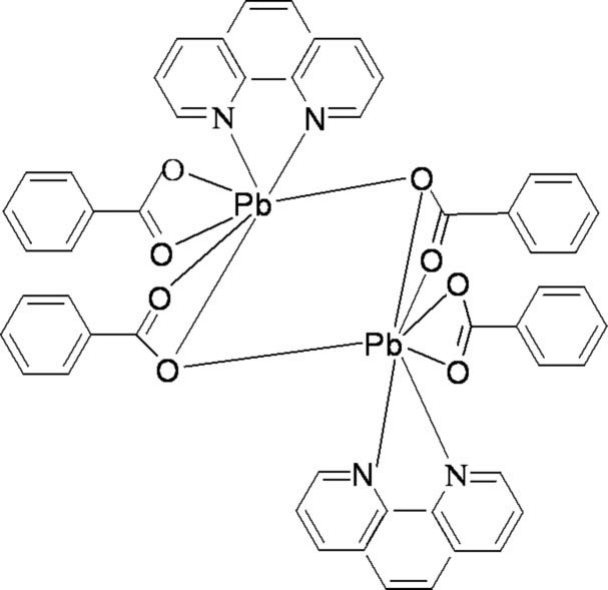


## Experimental

### 

#### Crystal data

[Pb_2_(C_7_H_5_O_2_)_4_(C_12_H_8_N_2_)_2_]
*M* *_r_* = 1259.23
Triclinic, 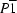

*a* = 9.011 (3) Å
*b* = 10.923 (3) Å
*c* = 11.920 (4) Å
α = 83.760 (3)°
β = 87.626 (3)°
γ = 71.601 (3)°
*V* = 1106.6 (6) Å^3^
*Z* = 1
Mo *K*α radiation
μ = 7.66 mm^−1^
*T* = 293 K
0.28 × 0.26 × 0.24 mm

#### Data collection

Bruker APEXII CCD diffractometer
Absorption correction: multi-scan (*SADABS*; Bruker, 2004 ▶) *T*_min_ = 0.223, *T*_max_ = 0.261
7969 measured reflections
4059 independent reflections
3296 reflections with *I* > 2σ(*I*)
*R*_int_ = 0.041

#### Refinement

*R*[*F* ^2^ > 2σ(*F* ^2^)] = 0.034
*wR*(*F* ^2^) = 0.069
*S* = 0.96
4059 reflections
298 parameters
H-atom parameters constrained
Δρ_max_ = 1.95 e Å^−3^
Δρ_min_ = −1.35 e Å^−3^

### 

Data collection: *APEX2* (Bruker, 2004 ▶); cell refinement: *SAINT* (Bruker, 2004 ▶); data reduction: *SAINT*; program(s) used to solve structure: *SHELXS97* (Sheldrick, 2008 ▶); program(s) used to refine structure: *SHELXL97* (Sheldrick, 2008 ▶); molecular graphics: *DIAMOND* (Brandenburg & Putz, 2005 ▶); software used to prepare material for publication: *SHELXTL* (Sheldrick, 2008 ▶).

## Supplementary Material

Crystal structure: contains datablocks I, global. DOI: 10.1107/S160053681101840X/wm2486sup1.cif

Structure factors: contains datablocks I. DOI: 10.1107/S160053681101840X/wm2486Isup2.hkl

Additional supplementary materials:  crystallographic information; 3D view; checkCIF report

## Figures and Tables

**Table 1:** Selected bond lengths (Å)

Pb1—O4	2.394 (4)
Pb1—N1	2.578 (5)
Pb1—O1	2.584 (4)
Pb1—N2	2.703 (5)
Pb1—O2	2.723 (5)
Pb1—O3	2.788 (5)
Pb1—O3^i^	2.924 (5)

Symmetry code: (i) 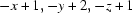.

**Fig. 1.**
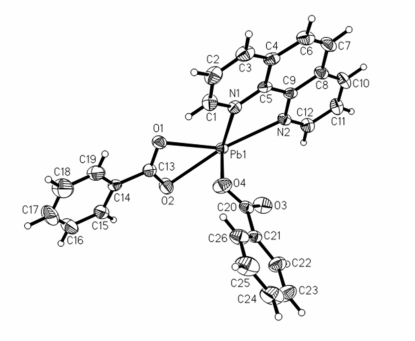
The asymmetric unit in the structure of the title complex, showing the atom-labeling scheme. Displacement ellipsoids are drawn at the 30% probability level.

**Fig. 2.**
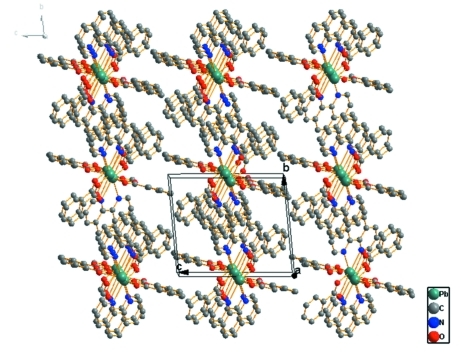
View approximately along the a axis, showing the three-dimensional framework structure in the title complex.

## supplementary crystallographic information

### Comment

Lead(II) compounds have been increasingly studied owing to their interesting physical and chemical properties (Fan *et al.*, 2006; Hu *et al.*, 2011). In order to extend our investigations in this field, we crystallised the lead(II) title compound [Pb_2_(C_7_H_5_O_2_)_4_(C_12_H_8_N_2_)_2_], and report its structure here.

The asymmetric unit of the title complex (Fig. 1) contains one Pb^2+^ ion, two benzoate anions, and one 1,10-phenanthroline ligand. The Pb^2+^ ion is seven-coordinated by five O atoms from three benzoate ligands and by two N atoms from 1,10-phenanthroline. The coordination environment around the Pb^2+^ ion may be described as a distorted mono-capped trigonal prism. Two adjacent Pb^II^ complexes are connected by two bridging benzoate anions to generate a centrosymmetric dinuclear unit. The benzoate anions adopt two kinds of coordination modes, *viz.* a bidentate chelating and a tridentate bridging-chelating mode.

The Pb—N and Pb—O bond lengths range between 2.578 (5)–2.703 (5) Å and 2.394 (4)–2.924 (5) Å, respectively. These values are in good agreement with those reported for other Pb(II)—O and Pb(II)—N donor complexes (Fan *et al.*, 2006; Hu *et al.*, 2011).

In the crystal structure, π—π stacking interactions between adjacent 1,10-phenanthroline ligands [centroid—centroid distance = 3.617 (4) Å] are observed. Furthermore, adjacent benzene rings from benzoate anions are also involved in π—π stacking interactions [centroid—centroid distance = 4.083 (3) Å]. π—π stacking interactions between adjacent 1,10-phenanthroline ligands and benzene rings from benzoate anions [centroid—centroid distance = 3.945 (4) Å] are also observed. These interactions of the discrete neutral molecules lead to a three-dimensional supramolecular framework (Fig. 2).

### Experimental

A mixture of Pb(CH_3_COO)_2_^.^3H_2_O (0.20 g, 0.54 mmol), benzoic acid (0.12 g, 1.0 mmol), 1,10-phenanthroline (0.11 g, 0.54 mmol), NaOH (0.04 g, 1.0 mmol), and water (10 ml) was stirred at room temperature for 15 min, and then sealed in a 25 ml Teflon-lined, stainless-steel Parr autoclave. The autoclave was heated at 433 K for 3 d. Upon cooling, the solution contained single crystals of the title complex in *ca* 80% yield. Anal./calc. for C_26_H_18_N_2_O_4_Pb: C, 49.60; H, 2.88; N, 4.45; found: C, 49.43; H, 3.07; N, 4.13.

### Refinement

The carbon-bound H atoms were placed in geometrically idealized positions, with C–H = 0.93 Å, and constrained to ride on their respective parent atoms, with *U*_iso_(H) = 1.2 *U*_eq_(C). The highest peak and the deepest hole in the final difference map are 0.98 Å and 0.89 Å, respectively, from Pb1.

### Crystal data

**Table:**

[Pb_2_(C_7_H_5_O_2_)_4_(C_12_H_8_N_2_)_2_]	*Z* = 1
*M**_r_* = 1259.23	*F*(000) = 604
Triclinic, *P*1	*D*_x_ = 1.889 Mg m^−^^3^
Hall symbol: -P 1	Mo *K*α radiation, λ = 0.71073 Å
*a* = 9.011 (3) Å	Cell parameters from 3450 reflections
*b* = 10.923 (3) Å	θ = 2.4–24.1°
*c* = 11.920 (4) Å	µ = 7.66 mm^−^^1^
α = 83.760 (3)°	*T* = 293 K
β = 87.626 (3)°	Block, colorless
γ = 71.601 (3)°	0.28 × 0.26 × 0.24 mm
*V* = 1106.6 (6) Å^3^	

### Data collection

**Table:**

Bruker APEXII CCD diffractometer	4059 independent reflections
Radiation source: fine-focus sealed tube	3296 reflections with *I* > 2σ(*I*)
graphite	*R*_int_ = 0.041
φ and ω scans	θ_max_ = 25.5°, θ_min_ = 2.4°
Absorption correction: multi-scan (*SADABS*; Bruker, 2004)	*h* = −10→10
*T*_min_ = 0.223, *T*_max_ = 0.261	*k* = −12→13
7969 measured reflections	*l* = −14→14

### Refinement

**Table:**

Refinement on *F*^2^	Primary atom site location: structure-invariant direct methods
Least-squares matrix: full	Secondary atom site location: difference Fourier map
*R*[*F*^2^ > 2σ(*F*^2^)] = 0.034	Hydrogen site location: inferred from neighbouring sites
*wR*(*F*^2^) = 0.069	H-atom parameters constrained
*S* = 0.96	*w* = 1/[σ^2^(*F*_o_^2^) + (0.0271*P*)^2^] where *P* = (*F*_o_^2^ + 2*F*_c_^2^)/3
4059 reflections	(Δ/σ)_max_ = 0.001
298 parameters	Δρ_max_ = 1.95 e Å^−^^3^
0 restraints	Δρ_min_ = −1.35 e Å^−^^3^

### Special details

**Table:**

Geometry. All e.s.d.'s (except the e.s.d. in the dihedral angle between two l.s. planes) are estimated using the full covariance matrix. The cell e.s.d.'s are taken into account individually in the estimation of e.s.d.'s in distances, angles and torsion angles; correlations between e.s.d.'s in cell parameters are only used when they are defined by crystal symmetry. An approximate (isotropic) treatment of cell e.s.d.'s is used for estimating e.s.d.'s involving l.s. planes.
Refinement. Refinement of *F*^2^ against ALL reflections. The weighted *R*-factor *wR* and goodness of fit *S* are based on *F*^2^, conventional *R*-factors *R* are based on *F*, with *F* set to zero for negative *F*^2^. The threshold expression of *F*^2^ > σ(*F*^2^) is used only for calculating *R*-factors(gt) *etc*. and is not relevant to the choice of reflections for refinement. *R*-factors based on *F*^2^ are statistically about twice as large as those based on *F*, and *R*- factors based on ALL data will be even larger.

### Fractional atomic coordinates and isotropic or equivalent isotropic displacement parameters (Å^2^)

**Table:**

	*x*	*y*	*z*	*U*_iso_*/*U*_eq_	
Pb1	0.24985 (3)	0.97230 (2)	0.484550 (19)	0.03770 (9)	
C3	0.1241 (8)	0.5411 (7)	0.4132 (6)	0.060 (2)	
H3	0.0979	0.4679	0.4009	0.072*	
C2	0.0639 (8)	0.6531 (8)	0.3469 (7)	0.064 (2)	
H2	−0.0042	0.6577	0.2890	0.077*	
C1	0.1049 (8)	0.7616 (7)	0.3660 (6)	0.0556 (18)	
H1	0.0621	0.8384	0.3202	0.067*	
N1	0.2020 (5)	0.7600 (5)	0.4465 (4)	0.0415 (12)	
C5	0.2638 (7)	0.6483 (6)	0.5128 (5)	0.0383 (14)	
C9	0.3737 (7)	0.6449 (6)	0.5979 (5)	0.0392 (14)	
N2	0.4171 (6)	0.7506 (5)	0.6046 (4)	0.0414 (12)	
C12	0.5255 (8)	0.7451 (6)	0.6789 (6)	0.0515 (17)	
H12	0.5580	0.8175	0.6821	0.062*	
C11	0.5919 (8)	0.6347 (7)	0.7520 (6)	0.062 (2)	
H11	0.6669	0.6340	0.8036	0.074*	
C10	0.5474 (8)	0.5294 (7)	0.7478 (6)	0.0603 (19)	
H10	0.5890	0.4562	0.7981	0.072*	
C8	0.4388 (8)	0.5296 (6)	0.6680 (6)	0.0484 (17)	
C7	0.3938 (9)	0.4183 (6)	0.6538 (7)	0.0591 (19)	
H7	0.4332	0.3431	0.7023	0.071*	
C6	0.2968 (9)	0.4201 (7)	0.5725 (7)	0.060 (2)	
H6	0.2746	0.3444	0.5624	0.072*	
C4	0.2253 (7)	0.5353 (6)	0.5000 (5)	0.0460 (16)	
O1	0.0004 (5)	1.0690 (4)	0.3639 (4)	0.0515 (11)	
O2	0.1272 (6)	1.2117 (5)	0.3714 (4)	0.0702 (15)	
C13	0.0280 (8)	1.1731 (6)	0.3290 (6)	0.0466 (16)	
C14	−0.0619 (7)	1.2544 (6)	0.2281 (5)	0.0417 (15)	
C19	−0.1491 (8)	1.2081 (8)	0.1642 (6)	0.068 (2)	
H19	−0.1557	1.1250	0.1825	0.081*	
C15	−0.0538 (8)	1.3785 (7)	0.1983 (6)	0.0580 (19)	
H15	0.0068	1.4112	0.2406	0.070*	
C18	−0.2279 (11)	1.2843 (12)	0.0720 (8)	0.104 (3)	
H18	−0.2879	1.2521	0.0287	0.125*	
C17	−0.2193 (12)	1.4049 (12)	0.0435 (8)	0.108 (4)	
H17	−0.2718	1.4546	−0.0197	0.130*	
C16	−0.1348 (11)	1.4533 (9)	0.1065 (7)	0.086 (3)	
H16	−0.1312	1.5372	0.0880	0.103*	
O3	0.5456 (6)	0.9341 (5)	0.3871 (4)	0.0672 (14)	
O4	0.3436 (6)	0.9290 (5)	0.2975 (4)	0.0689 (14)	
C20	0.4843 (8)	0.9206 (6)	0.3015 (6)	0.0436 (15)	
C21	0.5768 (7)	0.8906 (5)	0.1958 (5)	0.0410 (15)	
C26	0.5161 (9)	0.8558 (7)	0.1053 (6)	0.0612 (19)	
H26	0.4149	0.8506	0.1100	0.073*	
C25	0.5999 (13)	0.8290 (8)	0.0091 (7)	0.089 (3)	
H25	0.5563	0.8068	−0.0519	0.106*	
C22	0.7265 (8)	0.8974 (7)	0.1879 (6)	0.067 (2)	
H22	0.7698	0.9208	0.2485	0.080*	
C23	0.8124 (11)	0.8697 (9)	0.0907 (10)	0.097 (3)	
H23	0.9136	0.8749	0.0856	0.116*	
C24	0.7511 (14)	0.8349 (9)	0.0027 (8)	0.100 (4)	
H24	0.8105	0.8150	−0.0623	0.120*	

### Atomic displacement parameters (Å^2^)

**Table:**

	*U*^11^	*U*^22^	*U*^33^	*U*^12^	*U*^13^	*U*^23^
Pb1	0.04097 (14)	0.03437 (14)	0.04007 (15)	−0.01488 (10)	0.00285 (9)	−0.00557 (10)
C3	0.060 (5)	0.050 (5)	0.081 (6)	−0.027 (4)	0.015 (4)	−0.028 (4)
C2	0.059 (5)	0.068 (5)	0.074 (5)	−0.028 (4)	−0.009 (4)	−0.023 (4)
C1	0.058 (4)	0.053 (5)	0.059 (5)	−0.017 (4)	−0.007 (4)	−0.012 (4)
N1	0.041 (3)	0.038 (3)	0.049 (3)	−0.015 (2)	0.002 (2)	−0.012 (3)
C5	0.042 (3)	0.034 (3)	0.044 (4)	−0.017 (3)	0.015 (3)	−0.014 (3)
C9	0.041 (3)	0.036 (4)	0.042 (4)	−0.015 (3)	0.014 (3)	−0.009 (3)
N2	0.053 (3)	0.033 (3)	0.039 (3)	−0.015 (3)	0.008 (2)	−0.007 (2)
C12	0.058 (4)	0.044 (4)	0.055 (4)	−0.018 (3)	−0.002 (3)	−0.011 (3)
C11	0.064 (5)	0.055 (5)	0.060 (5)	−0.010 (4)	−0.011 (4)	−0.001 (4)
C10	0.078 (5)	0.045 (4)	0.048 (4)	−0.008 (4)	−0.007 (4)	0.007 (3)
C8	0.057 (4)	0.036 (4)	0.050 (4)	−0.013 (3)	0.015 (3)	−0.005 (3)
C7	0.074 (5)	0.034 (4)	0.067 (5)	−0.015 (4)	0.014 (4)	−0.003 (4)
C6	0.071 (5)	0.037 (4)	0.078 (6)	−0.023 (4)	0.026 (4)	−0.021 (4)
C4	0.047 (4)	0.041 (4)	0.059 (4)	−0.023 (3)	0.014 (3)	−0.019 (3)
O1	0.057 (3)	0.040 (3)	0.058 (3)	−0.017 (2)	0.003 (2)	−0.001 (2)
O2	0.072 (3)	0.063 (3)	0.082 (4)	−0.034 (3)	−0.032 (3)	0.012 (3)
C13	0.047 (4)	0.037 (4)	0.054 (4)	−0.011 (3)	0.007 (3)	−0.008 (3)
C14	0.040 (4)	0.050 (4)	0.038 (4)	−0.018 (3)	0.008 (3)	−0.006 (3)
C19	0.073 (5)	0.091 (6)	0.053 (5)	−0.046 (5)	0.000 (4)	−0.005 (4)
C15	0.063 (5)	0.057 (5)	0.054 (4)	−0.021 (4)	0.004 (4)	−0.001 (4)
C18	0.122 (8)	0.155 (11)	0.062 (6)	−0.081 (8)	−0.024 (5)	0.000 (7)
C17	0.123 (9)	0.143 (10)	0.057 (6)	−0.050 (8)	−0.034 (6)	0.034 (7)
C16	0.094 (7)	0.084 (6)	0.068 (6)	−0.023 (5)	0.004 (5)	0.024 (5)
O3	0.093 (4)	0.074 (4)	0.046 (3)	−0.038 (3)	0.002 (3)	−0.019 (3)
O4	0.069 (3)	0.093 (4)	0.050 (3)	−0.032 (3)	0.018 (2)	−0.017 (3)
C20	0.058 (4)	0.027 (3)	0.048 (4)	−0.017 (3)	0.004 (3)	−0.005 (3)
C21	0.051 (4)	0.030 (3)	0.042 (4)	−0.013 (3)	0.012 (3)	−0.006 (3)
C26	0.081 (5)	0.061 (5)	0.047 (4)	−0.029 (4)	0.014 (4)	−0.013 (4)
C25	0.129 (9)	0.086 (7)	0.056 (5)	−0.038 (6)	0.025 (5)	−0.027 (5)
C22	0.060 (5)	0.075 (5)	0.064 (5)	−0.021 (4)	0.017 (4)	−0.012 (4)
C23	0.073 (6)	0.094 (7)	0.114 (8)	−0.018 (5)	0.044 (6)	−0.010 (7)
C24	0.137 (10)	0.069 (6)	0.074 (7)	−0.007 (6)	0.060 (7)	−0.017 (5)

### Geometric parameters (Å, °)

**Table:**

Pb1—O4	2.394 (4)	O1—C13	1.263 (7)
Pb1—N1	2.578 (5)	O1—Pb1^ii^	2.946 (4)
Pb1—O1	2.584 (4)	O2—C13	1.247 (8)
Pb1—N2	2.703 (5)	C13—C14	1.512 (9)
Pb1—O2	2.723 (5)	C13—Pb1^ii^	3.874 (6)
Pb1—O3	2.788 (5)	C14—C19	1.356 (9)
Pb1—O3^i^	2.924 (5)	C14—C15	1.387 (9)
C3—C2	1.350 (10)	C19—C18	1.377 (11)
C3—C4	1.390 (9)	C19—H19	0.9300
C3—H3	0.9300	C15—C16	1.376 (10)
C2—C1	1.390 (9)	C15—H15	0.9300
C2—H2	0.9300	C18—C17	1.350 (13)
C1—N1	1.320 (8)	C18—H18	0.9300
C1—H1	0.9300	C17—C16	1.345 (12)
N1—C5	1.350 (7)	C17—H17	0.9300
C5—C4	1.408 (8)	C16—H16	0.9300
C5—C9	1.436 (8)	O3—C20	1.224 (7)
C9—N2	1.342 (7)	O3—Pb1^i^	2.923 (5)
C9—C8	1.404 (8)	O4—C20	1.244 (7)
N2—C12	1.329 (8)	C20—C21	1.490 (8)
C12—C11	1.389 (9)	C21—C26	1.370 (9)
C12—H12	0.9300	C21—C22	1.374 (9)
C11—C10	1.339 (10)	C26—C25	1.353 (10)
C11—H11	0.9300	C26—H26	0.9300
C10—C8	1.392 (9)	C25—C24	1.383 (13)
C10—H10	0.9300	C25—H25	0.9300
C8—C7	1.425 (9)	C22—C23	1.374 (11)
C7—C6	1.326 (10)	C22—H22	0.9300
C7—H7	0.9300	C23—C24	1.348 (13)
C6—C4	1.429 (9)	C23—H23	0.9300
C6—H6	0.9300	C24—H24	0.9300
			
O4—Pb1—N1	73.03 (16)	C6—C7—H7	119.4
O4—Pb1—O1	76.92 (16)	C8—C7—H7	119.4
N1—Pb1—O1	80.92 (15)	C7—C6—C4	121.7 (6)
O4—Pb1—N2	100.85 (16)	C7—C6—H6	119.1
N1—Pb1—N2	62.22 (16)	C4—C6—H6	119.1
O1—Pb1—N2	141.48 (15)	C3—C4—C5	117.5 (6)
O4—Pb1—O2	79.49 (17)	C3—C4—C6	123.6 (6)
N1—Pb1—O2	127.45 (15)	C5—C4—C6	118.9 (6)
O1—Pb1—O2	49.27 (13)	C13—O1—Pb1	95.5 (4)
N2—Pb1—O2	169.20 (13)	C13—O1—Pb1^ii^	129.6 (4)
O4—Pb1—O3	48.70 (15)	Pb1—O1—Pb1^ii^	103.74 (14)
N1—Pb1—O3	100.12 (14)	C13—O2—Pb1	89.4 (4)
O1—Pb1—O3	120.86 (14)	O2—C13—O1	124.0 (6)
N2—Pb1—O3	78.88 (14)	O2—C13—C14	117.9 (6)
O2—Pb1—O3	93.68 (15)	O1—C13—C14	118.1 (6)
O4—Pb1—O3^i^	113.45 (15)	C19—C14—C15	118.7 (7)
N1—Pb1—O3^i^	140.87 (15)	C19—C14—C13	121.5 (6)
O1—Pb1—O3^i^	137.97 (14)	C15—C14—C13	119.8 (6)
N2—Pb1—O3^i^	78.83 (15)	C14—C19—C18	119.9 (8)
O2—Pb1—O3^i^	91.08 (14)	C14—C19—H19	120.1
O3—Pb1—O3^i^	66.84 (16)	C18—C19—H19	120.1
C2—C3—C4	120.0 (6)	C16—C15—C14	120.3 (7)
C2—C3—H3	120.0	C16—C15—H15	119.8
C4—C3—H3	120.0	C14—C15—H15	119.8
C3—C2—C1	119.3 (7)	C17—C18—C19	121.0 (9)
C3—C2—H2	120.3	C17—C18—H18	119.5
C1—C2—H2	120.3	C19—C18—H18	119.5
N1—C1—C2	122.8 (7)	C16—C17—C18	120.1 (9)
N1—C1—H1	118.6	C16—C17—H17	120.0
C2—C1—H1	118.6	C18—C17—H17	120.0
Pb1—C1—H1	78.0	C17—C16—C15	120.0 (9)
Pb1^ii^—C1—H1	66.1	C17—C16—H16	120.0
C1—N1—C5	118.4 (5)	C15—C16—H16	120.0
C1—N1—Pb1	119.7 (4)	C20—O3—Pb1	85.0 (4)
C5—N1—Pb1	121.7 (4)	C20—O3—Pb1^i^	155.1 (4)
N1—C5—C4	122.0 (6)	Pb1—O3—Pb1^i^	113.16 (16)
N1—C5—C9	118.6 (5)	C20—O4—Pb1	103.6 (4)
C4—C5—C9	119.3 (6)	O3—C20—O4	122.8 (6)
N2—C9—C8	121.7 (6)	O3—C20—C21	121.1 (6)
N2—C9—C5	118.8 (5)	O4—C20—C21	116.1 (6)
C8—C9—C5	119.4 (6)	C26—C21—C22	118.7 (6)
C12—N2—C9	118.9 (5)	C26—C21—C20	121.5 (6)
C12—N2—Pb1	123.4 (4)	C22—C21—C20	119.8 (6)
C9—N2—Pb1	117.2 (4)	C25—C26—C21	121.5 (8)
N2—C12—C11	122.0 (6)	C25—C26—H26	119.2
N2—C12—H12	119.0	C21—C26—H26	119.2
C11—C12—H12	119.0	C26—C25—C24	119.2 (9)
Pb1—C12—H12	80.8	C26—C25—H25	120.4
C10—C11—C12	119.7 (7)	C24—C25—H25	120.4
C10—C11—H11	120.2	C23—C22—C21	120.1 (8)
C12—C11—H11	120.2	C23—C22—H22	120.0
C11—C10—C8	119.9 (6)	C21—C22—H22	120.0
C11—C10—H10	120.0	C24—C23—C22	120.4 (9)
C8—C10—H10	120.0	C24—C23—H23	119.8
C10—C8—C9	117.7 (6)	C22—C23—H23	119.8
C10—C8—C7	122.9 (6)	C23—C24—C25	120.1 (8)
C9—C8—C7	119.4 (6)	C23—C24—H24	120.0
C6—C7—C8	121.1 (7)	C25—C24—H24	120.0

Symmetry codes: (i) −*x*+1, −*y*+2, −*z*+1; (ii) −*x*, −*y*+2, −*z*+1.
